# Supplementary material for: The genome sequence of the daisy, Bellis perennis L. (Asterales: Asteraceae)
Source: Wellcome Open Res. 2026 Oct 5;11:798. [Version 1] doi: 10.12688/wellcomeopenres.28039.1 (PMC13653108; doi:10.12688/wellcomeopenres.28039.1)
Supplement: Peer review history

# Reviewer report: Reviewer response for version 1

**DOI:** 10.21956/wellcomeopenres.30845.r172640

**Published:** 2026-10-09

**Authors:** Ruslan Kalendar

**Affiliations:** 1 University of Helsinki, Helsinki, Finland

The authors present a phased assembly of the diploid (2n = 18) genome of the daisy, *Bellis perennis*, generated within the Darwin Tree of Life project from PacBio HiFi (62.67 Gb; Sequel II/IIe), Hi-C (256.03 Gb) and RNA-seq data. Haplotype 1 (1,602.72 Mb) has been curated to chromosome level: nine pseudomolecules contain 93.94% of the sequence, with contig N50 4.66 Mb, scaffold N50 176.96 Mb, QV 65.8 and BUSCO completeness of 95.7%. Haplotype 2 (1,503.20 Mb) remains at scaffold level. The mitochondrial (262.08 kb) and plastid (151.87 kb) genomes were also assembled.

 This is a valuable resource: it is a large (1C = 1.74 Gb), highly repetitive (74%), and highly heterozygous (2.38%) genome, and the assembly is chromosome-scale.  

 Q1. The text states that “the Hi-C map reveals that the two haplotypes are highly divergent … especially Chromosome 5” and that “the Hi-C signal is poor for some of the chromosomes in the haplotype 2 assembly,” but only the haplotype 1 map is shown (Figure 3), and a Hi-C contact map is not the appropriate tool for measuring divergence between haplotypes. In Figure 3, chromosomes 5 and 7 show a clear anti-diagonal (“bow-tie”) signal within the chromosome.

 This pattern is typical of a large inversion that is heterozygous in the sequenced individual (Hi-C reads from the inverted haplotype mapping to haplotype 1). Please (i) show the haplotype 2 Hi-C map (e.g., as an extended-data figure), (ii) provide a whole-genome alignment of the two haplotypes (e.g., minimap2 dot plots or SyRI), and (iii) state explicitly whether heterozygous inversions are present, with approximate coordinates and sizes. Such structural variants are of direct interest to users of this reference.

 Q2. Manual curation “reduced the total assembly length by 7.0%”. For the combined haplotypes (3.1-3.3 Gb), this corresponds to approximately 220-230 Mb, which is far more than is usually removed during curation.

 Haplotype 2 is 100 Mb shorter than haplotype 1 (1,503.20 vs 1,602.72 Mb; 86% vs 92% of the flow-cytometry estimate), yet its scaffold N50 (164.73 Mb) and longest scaffold (203.06 Mb) are chromosome-scale.  

 Q2. The paper gives no coverage figure of its own and instead cites “24× genome coverage” from the NCBI record. From the data, 62.67 Gb corresponds to 36× of the 1C value (1,740 Mb), that is, only 18× per haplotype, in line with the GenomeScope haploid k-mer coverage of 17.6. In the blob plot (Figure 6), the chromosomal scaffolds sit at only 13×.

 Q3. At a haploid k-mer coverage of 17.6×, the error and heterozygous peaks partly overlap (Figure 2), and the model fit was 94.84%. Therefore, the estimates of heterozygosity (2.38%) and repeat content (74.02%) are less robust than usual and should be presented as such. Since the Background mentions possible hybridization of horticultural forms with *B. sylvestris*, a Smudgeplot (from the same k-mer database) would be a cheap and useful confirmation of diploidy for this wild-collected individual.

 Q4. The text and Table 4 report eudicots_odb10 (n = 2,326; C = 95.7%, D = 8.1%), whereas the snail plot (Figure 5) uses eudicotyledons_odb12 (n = 2,805; C = 96.26%, D = 7.84%). Table 5 lists BUSCO 6.0.0 and 6.1.0 without saying which was used where. Please use a single, current dataset throughout, or explicitly report both. The duplicated fraction (8%) also deserves a comment. It may reflect the ancient whole-genome triplication at the base of the Asteraceae, but residual haplotypic duplication should be excluded, for example, by checking whether duplicated BUSCOs lie within chromosomes or on unplaced scaffolds.

 Q5. The chromosomes in Table 3 sum to 1,505.60 Mb, leaving 97.1 Mb unplaced. According to Figure 6, 745 scaffolds totalling 91.8 Mb are classified as “no-hit”. Most form a distinct cluster at GC =50% with coverage of 10-1,000×, which is characteristic of rDNA arrays or satellite repeats.

 Please briefly describe these scaffolds and what ASCC removed. Combined with the 140 Mb shortfall of haplotype 1 relative to flow cytometry (1,602.72 vs 1,740 Mb), this would give readers a realistic picture of which repeat classes are under-represented.

Are the datasets clearly presented in a usable and accessible format, and the assembly and annotation available in an appropriate subject-specific repository?

Yes

Are sufficient details of the sequencing and extraction, software used, and materials provided to allow replication by others?

Yes

Are the rationale for sequencing the genome and the species significance clearly described?

Yes

Are the protocols appropriate and is the work technically sound?

Yes

Reviewer Expertise:

plant genome bioinformatics

I confirm that I have read this submission and believe that I have an appropriate level of expertise to confirm that it is of an acceptable scientific standard.

# Reviewer report: Reviewer response for version 1

**Published:** 2026-10-05

**Authors:** Automated Benchmarking

**Affiliations:** 1 Wellcome Sanger Institute, Hinxton, UK

As this article is a Genome Note, Automated Benchmarking has been used to determine whether the reported assembly passes four defined checks established by the Earth BioGenome Project: the raw and assembled data are publicly available, the Earth BioGenome Project three-part metric is met, the k-mer completeness exceeds 90%, and the BUSCO score passes the stated threshold. For Bellis perennis (daBelPere1), all four checks passed. Data available: pass. ENA study PRJEB88870, raw-read accessions ERR14886565, ERR14886562, ERR14886563, ERR14886564, ERR14895980, and ERR14895981, and assembly accessions GCA_965601635.2 and GCA_965601805.2 are public. Earth BioGenome Project three-part metric: pass. Haplotype 1 is reported as 6.C.Q65, meeting the 6.C.Q40 reference standard. k-mer completeness: pass. The combined haplotype assemblies have 96.48% k-mer completeness, exceeding the Earth BioGenome Project target of 90%. BUSCO: pass. Haplotype 1, which is proposed as the reference assembly for this species, has 95.7% complete BUSCOs (87.6% single-copy and 8.1% duplicated). BUSCO software version: 6.0.0; lineage dataset: eudicots_odb10. It meets the plant-specific target of greater than 90% complete BUSCOs. This decision uses values reported in the Genome Note and public accession records.

Reviewer Expertise:

NA

I confirm that I have read this submission and believe that I have an appropriate level of expertise to confirm that it is of an acceptable scientific standard.
